# A Generalized Formula for Converting Chi-Square Tests to Effect Sizes for Meta-Analysis

**DOI:** 10.1371/journal.pone.0010059

**Published:** 2010-04-07

**Authors:** Michael S. Rosenberg

**Affiliations:** Center for Evolutionary Medicine and Informatics, The Biodesign Institute and School of Life Sciences, Arizona State University, Tempe, Arizona, United States of America; University of Liverpool, United Kingdom

## Abstract

The common formula used for converting a chi-square test into a correlation coefficient for use as an effect size in meta-analysis has a hidden assumption which may be violated in specific instances, leading to an overestimation of the effect size. A corrected formula is provided.

## Introduction

One of the fundamental concepts in systematic and comparative reviews such as meta-analysis is that of the effect size. An effect size is a statistical parameter that can be used to compare, on the same scale, the results of different studies in which a common effect of interest has been measured [1], [2]. In experimental studies, the effect size is a measurement of the response of the subjects to an experimental treatment relative to a control group. All effect size measures are a means of representing the results of primary research in a common way so that the results from individual studies can be compared and evaluated [1]. While a number of alternate metrics have been suggested for measuring effect size, including standardized mean differences and odds ratios [3], [4], historically, one of the more popular measures of effect has been the correlation coefficient [5], [6], [7], [8], [9], [10]. The correlation coefficient is widely used, easily interpretable, and has the added bonus of being easily determinable from other commonly used statistics such as *z*-scores, *t*-tests, *F*-statistics, and χ^2^ statistics [3], [11]. These conversions can only be performed for single, focused contrasts (*e.g.*, cases with a single degree of freedom), but otherwise follow simple equations. For example, the equation for converting a χ^2^ into a correlation [11], [12] is:
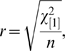
(1)where the χ^2^ value comes from a two-group contrasts (thus a single degree of freedom) and *n* is the total number of samples; the sign of the correlation needs to be determined from independent study of the contrast. (χ^2^ tests with more than one degree of freedom are unfocused omnibus tests, and require a much more complicated procedure for conversion to an effect size; see [13], [14], [15] for details). Equation (1) has been used to convert χ^2^ tests into a correlation coefficient for use as an effect size for 45 years [12]; unfortunately, this equation has an underlying, never-stated assumption which is sometimes violated, particularly for genetics studies: it assumes that the expected values from the χ^2^ test are equal for both groups.

## Results and Discussion

Recall that the χ^2^ is calculated simply as
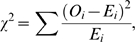
(2)where *O_i_* and *E_i_* are the observed and expected counts, respectively, for group *i*. Equation (1) assumes that the expected values for the two groups are equal, that is, *E*_1_ = *E*_2_ = *n*/2. For the vast majority of χ^2^ tests this assumption probably holds and equation (1) is correct. However, when this assumption is not met (*e.g.*, if the expected values are for a phenotypic ratio of 3∶1 from a standard Mendelian di-heterozygous dominant-recessive cross), equation (1) is incorrect and will overestimate the effect size (occasionally even producing “correlations” greater than one).

The problem is specifically found in the denominator of the equation. Rather than the sample size, the denominator should actually be the maximum possible χ^2^ value obtainable for that sample size and expected values:
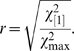
(3)


Written this way, the logic of the equation can be interpreted as *r* ^2^ being equal to the ratio of observed χ^2^ to the maximum possible χ^2^, a not atypical way of expressing the variance explained in a sums-of-squares framework. In the common case where one expects an equal distribution of observations among the two groups (*E*_1_ = *E*_2_ = *n*/2), this maximum possible value is equal to *n* (see Methods), making equation (1) correct.

On the other hand, if the expected values for the two groups are not the same, the maximum possible value is larger than *n*. Specifically, if the ratio of expectations among the two groups is *k*∶1, where *k* represents the larger group (*k*≥1), the maximum possible χ^2^ value is equal to *nk* (see Methods). The general form of the conversion of a χ^2^ value to *r* should therefore be
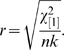
(4)


As would be expected, this generalized form simplifies to the commonly used equation when the expected ratio of observations is 1∶1 (*i.e., k* = 1).

As a simple example, imagine a genetic cross of two heterozygous individuals for a pair of alleles with a simple dominant-recessive relationship. The expectation is a phenotypic ratio of 3∶1, in favor of the dominant phenotype. With 22 total offspring, the expected counts are 16.5 and 5.5, respectively. The actual observation is almost the reverse, with 16 found to have the recessive phenotype and only 6 having the dominant phenotype. The χ^2^ value for this cross is 26.7 (some additional factor is clearly skewing the expected phenotypic ratio). Using the traditional transformation from equation (1), one would determine an effect size *r* = 1.10, a value outside the acceptable range of *r*, impossible to interpret or use in a summary analysis (which generally requires use of Fisher's *z*-transform). Taking into account the adjusted formula due to the assumption of unequal expectations, we instead find an effect size of *r* = 0.64, still quite strong, but logically interpretable and meaningful.

It is difficult to determine how often, if ever, equation (1) has been misapplied, because one could not simply go through published meta-analyses looking for χ^2^ conversions, but would rather have to go back to the original studies used in each meta-analysis to determine how the χ^2^ test was actually calculated. Fortunately, the assumption of equal expectation among groups likely holds for the majority of χ^2^ tests. However, those who work in fields or encounter data where this assumption is not true (e.g., heredity experiments), need to be aware of the alternate formulation to use for their specific work, particularly as meta-analysis and other statistics that make use of effect size continue to become more common [16].

## Methods

### Derivation of maximum possible χ^2^ value for two groups when the expectation is an equal distribution of counts among the groups

The χ^2^ value is calculated as
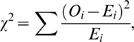
(5)where *O_i_* and *E_i_* are the observed and expected values for the *i* ^th^ group. In a χ^2^ test with only two groups with *n* total observations, if the expectation is an equal distribution of observations among groups, the expected value of each of the two groups will be *n*/2. The maximum possible χ^2^ value will be obtained if one group contains all of the observations and the other group contains zero observations, thus
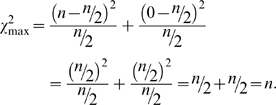
(6)


The maximum possible χ^2^ value for a two group case where the expected distribution of outcomes is evenly distributed among the two groups is simply the total sample size.

### Derivation of maximum possible χ^2^ value for two groups when the expectation is not an equal distribution of counts among the groups

If the expectation among the two groups is not equal, we have to make the following change. The expected ratio between the two groups will be *k*∶1, where *k* is how many times larger one group is expected to be relative to the other (*k*≥1). In this case the expected values are *nk*/(*k*+1) and *n*/(*k*+1), respectively (Note that if *k* = 1, we have an expected ratio of 1∶1, leading both expectations to equal *n*/2 and the special case described above). Given these new expectations, the maximum possible χ^2^ value will be when the group with the “*nk*” expectation (the larger expectation) contains 0 observations and the other group (the smaller expectation) contains all *n* observations. In this case, the maximum possible χ^2^ value becomes:
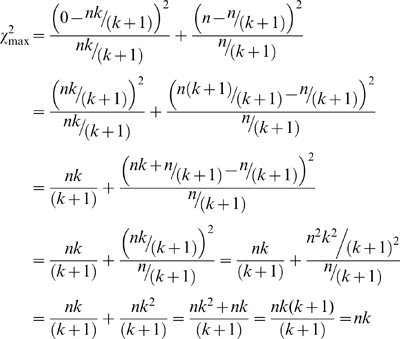
(7)


Again, if the expectation is even, *k* = 1, and the maximum χ^2^ value is simply *n*.
